# Novel, Synergistic Antifungal Combinations that Target Translation Fidelity

**DOI:** 10.1038/srep16700

**Published:** 2015-11-17

**Authors:** Elena Moreno-Martinez, Cindy Vallieres, Sara L. Holland, Simon V. Avery

**Affiliations:** 1 School of Life Sciences, University of Nottingham University Park, Nottingham NG7 2RD, UK.

## Abstract

There is an unmet need for new antifungal or fungicide treatments, as resistance to existing treatments grows. Combination treatments help to combat resistance. Here we develop a novel, effective target for combination antifungal therapy. Different aminoglycoside antibiotics combined with different sulphate-transport inhibitors produced strong, synergistic growth-inhibition of several fungi. Combinations decreased the respective MICs by ≥8-fold. Synergy was suppressed in yeast mutants resistant to effects of sulphate-mimetics (like chromate or molybdate) on sulphate transport. By different mechanisms, aminoglycosides and inhibition of sulphate transport cause errors in mRNA translation. The mistranslation rate was stimulated up to 10-fold when the agents were used in combination, consistent with this being the mode of synergistic action. A range of undesirable fungi were susceptible to synergistic inhibition by the combinations, including the human pathogens *Candida albicans, C. glabrata* and *Cryptococcus neoformans,* the food spoilage organism *Zygosaccharomyces bailii* and the phytopathogens *Rhizoctonia solani* and *Zymoseptoria tritici.* There was some specificity as certain fungi were unaffected. There was no synergy against bacterial or mammalian cells. The results indicate that translation fidelity is a promising new target for combinatorial treatment of undesirable fungi, the combinations requiring substantially decreased doses of active components compared to each agent alone.

Diverse fungi are considered undesirable, including human and animal (livestock) pathogens, crop pathogens, food spoilage fungi and fungi causing deterioration of materials or indoor air quality. Fungal pathogens cause as many or more deaths than drug-resistant tuberculosis and malaria^1^. Among the most important opportunistic fungal pathogens of humans are *Candida* spp. the fourth most common cause of nosocomial infection, with a mortality rate close to 34%^2^. Other common fungal pathogens include *Aspergillus*, *Cryptococcus* and *Pneumocystis* spp. In addition, fungi can be devastating pathogens of food crops and other plants. Reflecting this, the global fungicide market is worth more than $7 billion^3^,^4^. Up to one third of all foods is spoiled by fungi, the majority of this being stored agricultural products^5^,^6^. A range of antifungals or fungicides have been developed to counter such undesirable fungi^4^,^7^. Drugs used for treatment of fungal infections include the polyenes, azoles and echinocandins. A wider range of fungicides have been approved for use against phytopathogens, including metal-based compounds. Certain antibiotics are still approved as fungicides in some countries despite concerns about spread of antibiotic resistance.

Evolution of resistance to antifungals and particularly fungicides is a growing problem, underscoring the urgent need for development of new effective treatments^4^,^7^. Combination treatments are attracting particular attention as a method of management. An advantage of such combinations is that they reduce the likelihood of resistance: evolution of resistance to more than one agent is much slower than with a single agent^8^,^9^. Such combinations can be particularly effective where they produce a synergistic action against the fungus. This allows lower doses of the agents to be used than if supplied singly, lessening potential concerns over non-specific toxicity or cost. For example, the minimal inhibitory concentration of the antifungal caspofungin could be decreased by up to 5-fold in combination with a cheap drug (chloroquine) with which there was synergistic inhibition^10^. Such strong synergies are not very common, but their detection is facilitated by screening approaches (chemical or biological) and/or the use of a good model system. The yeast *Saccharomyces cerevisiae* is widely adopted as a eukaryotic cell model of choice and has been applied to characterize the actions of antifungal drugs^10^,^11^ as well as a diverse range of other therapeutic compounds^12^,^13^,^14^,^15^.

In previous work with *S. cerevisiae* to characterise mode-of-action of the metal toxicant chromate, synergistic inhibition of yeast growth was observed when chromate was combined with the aminoglycoside antibiotic paromomycin^16^. For the purposes of that study, this result among others helped to establish that chromate treatment provokes errors in the process of mRNA translation during protein synthesis, producing toxic protein aggregates. For the purposes of the present study, we re-evaluate that observed synergy from the perspective of its antifungal potential. Whereas aminoglycosides are commonly used as antibacterials due to the sensitivity of prokaryotic ribosomes, at elevated doses they also target eukaryotic ribosomes and so can inhibit fungi, with some aminoglycosides used as fungicides^17^,^18^,^19^. As mentioned above, certain metal-based compounds have also been approved for crop protection. Here, first we exploit *S. cerevisiae* to elucidate alternative agents that can be combined synergistically with paromomcyin or chromate, lowering their effective concentrations, and we describe the mechanism of synergistic inhibition. Moreover, we show that the combinations are highly effective at inhibiting growth of certain major pathogenic and food-spoilage fungi; the subject of a GB (priority) patent application (25-03-2015). It is proposed that combination targeting of translation fidelity can offer an effective strategy for controlling fungal growth.

## Results

### Aminoglycosides combined with sulphate transport inhibitors produce synergistic inhibition of yeast growth

**Figure 1:**
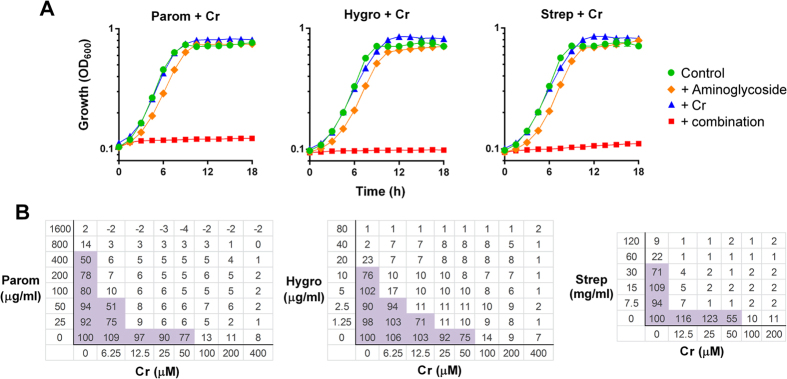
Aminoglycosides combined with chromate produce synergistic inhibition of yeast growth. (**A**) Growth of *S. cerevisiae* BY4743 was monitored continuously in YEPD broth supplemented as indicated. The agents and doses were: paromomycin (Parom), 200–250 μg ml^−1^; hygromycin B (Hygro), 10 μg ml^−1^; streptomycin (Strep), 30 mg ml^−1^ (the highest soluble dose attainable); chromate (Cr), 50 μM. Data shown are replicates from two independent cultures ± SEM where these are larger than the symbol dimensions. The data for each condition are representative of at least two independent experiments performed on different days. (**B**) Checkerboard assays with *S. cerevisiae* at the indicated concentrations, according to EUCAST procedure. The growth values within the boxes are percentages of control growth (OD) determined in the absence of aminoglycoside or Cr. Shaded boxes indicate ≥50% of control growth.

The aminoglycoside paromomycin and the toxic metal chromate were each supplied at sub-inhibitory concentrations. When combined, these produced almost complete inhibition of growth of the model yeast *S. cerevisiae* (Fig. 1A). Synergy is evident where the growth inhibition effect is significantly greater with the agents combined than by a simple product of their individual effects at the same doses. Synergy with Cr was not specific to paromomycin. Streptomycin and hygromycin as alternative aminoglycosides also both produced strong synergistic growth inhibition when combined with Cr (Fig. 1A). Checkerboard analysis showed that combinations decreased the MICs of the individual agents by ≥8-fold in the cases of either paromomycin or streptomycin with Cr, and by ≥4-fold for hygromycin with Cr (Fig. 1B). Mean fractional inhibitory concentrations (FIC) for these combinations were calculated to be 0.17, 0.25 and 0.29 respectively; combinations are normally considered synergistic when the mean FIC is ≤0.50^20^.

**Figure 2:**
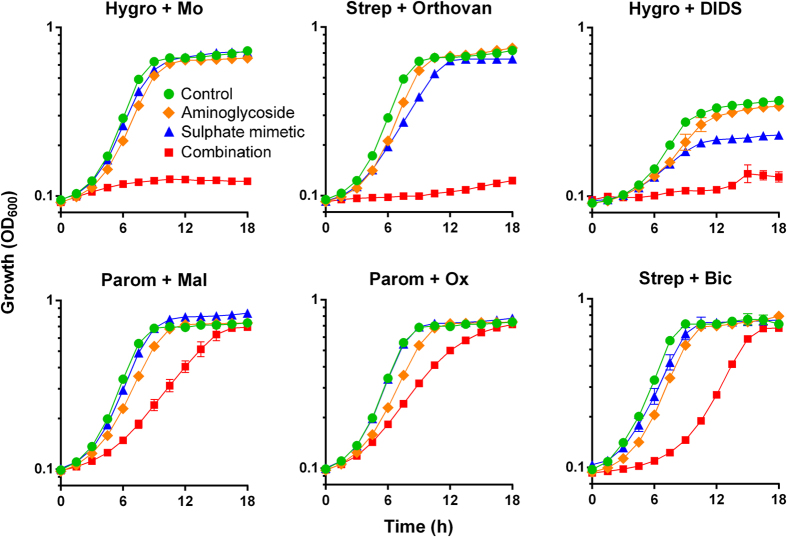
Aminoglycosides combined with sulphate transport inhibitors produce synergistic inhibition of yeast growth. Growth of *S. cerevisiae* BY4743 was monitored continuously in YEPD broth (or YNB broth for Hygro+DIDS, as DIDS formed precipitates in YEPD), supplemented with different aminoglycosides in combination with sulphate transport inhibitors (the most effective synergistic combinations are presented; other combinations are in Supplementary Fig. S1). Aminoglycoside doses were as for Fig. 1 except in combination with DIDS where hygromycin B was at 150 μg ml^−1^. The sulphate transport inhibitors were chromate (Cr), 50 μM; molybdate (Mo), 1 mM, orthovanadate (Orthovan), 1 mM; DIDS, 1 mM; malonate (Mal), 50 mM (the highest sub-inhibitory dose tested); oxalate (Ox), 5 mM (the highest soluble dose attainable); bicarbonate (Bic), 7.5 mM. Data shown are replicates from two independent cultures ± SEM where these are larger than the symbol dimensions. The data for each condition are representative of at least two independent experiments performed on different days.

The stimulation of mRNA mistranslation by Cr has been indicated previously to arise from interference with sulphate uptake, leading to a limitation for (biosynthesis of) sulphur containing amino acids^21^. Therefore we tested alternative agents reported to interfere with sulphate transport or metabolism^22^, each in combination with paromomycin, streptomycin or hygromycin. The combination giving the strongest effect with each sulphate transport inhibitor is presented (Fig. 2) with the other combinations in the supplementary material (Supplementary Fig. S1). Sodium molybdate produced synergistic inhibition of yeast growth with aminoglycosides, particularly hygromycin. A comparatively high molybdate dose was used (1 mM) as it is less toxic than chromate. At a similar concentration, sodium orthovanadate gave strong growth inhibition in combination with streptomycin (Fig. 2), with the effect a little less marked in combinations with paromomycin and particularly hygromycin (Supplementary Fig. S1). There was also synergy between hygromycin and the anion exchange inhibitor 4,4′-diisothio-cyanatostilbene-2,2′-disulphonate (DIDS) (Fig. 2). There was weaker evidence for synergistic growth inhibition with sodium-bicarbonate (most effective with streptomycin), -oxalate or -malonate (most effective with paromomycin). We found no evidence for synergy in yeast when aminoglycosides were combined with certain putative sulphate-transport inhibitors: selenate, thiosulphate, tungstate or tetrathionate (data not shown). The results showed that a number of alternative aminoglycosides in combination with a number of alternative sulphate transport inhibitors produce synergistic growth inhibition of yeast.

### Synergy is suppressed in mutants resistant to sulphate-transport inhibition

**Figure 3:**
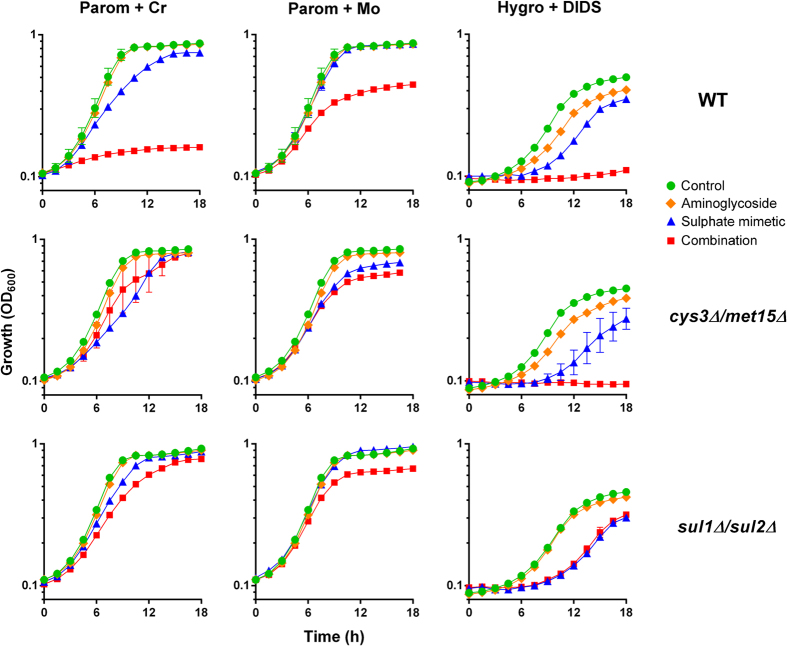
Synergy is suppressed in mutants resistant to sulphate-transport inhibition. Growth of wild type *S. cerevisiae* BY4741 or isogenic *cys3Δ/met15Δ* or *sul1Δ/sul2Δ* mutants was monitored continuously in YEPD or YNB (for Hygro+DIDS) broths. The appropriate doses of indicated agents used to test synergy were: 50 μg ml^−1^ paromomycin for wild type and *cys3Δ/met15Δ*, 300 μg ml^−1^ paromomycin for *sul1Δ/sul2Δ*, 150 μg ml^−1^ hygromycin B, 50 μM chromate, 1 mM molybdate, 1 mM DIDS. Data shown are replicates from two independent cultures ± SEM where these are larger than the symbol dimensions. The data for each condition are representative of at least two independent experiments performed on different days.

To test the hypothesis that the mechanism of synergy involved perturbation of sulphate supply, first we used an *S. cerevisiae cys3Δ/met15Δ* mutant. This mutant takes up cysteine and methionine directly from the growth medium, rather than synthesis in the cell from transported sulphate, so is resistant to effects of chromate on sulphate uptake and on translation error^21^. Supporting the hypothesis, synergistic growth inhibition by paromomycin and Cr was rescued in the *cys3Δ/met15Δ* mutant (Fig. 3). Synergistic growth inhibition by paromomycin combined with an alternative sulphate mimetic, molybdate, also was largely suppressed in the *cys3Δ/met15Δ* mutant (Fig. 3). In the case of hygromycin B combined with DIDS, a more general anion transport inhibitor, synergy seen in the wild type was not abolished in the mutant (Fig. 3). The results are consistent with inhibition of sulphate-transport being the relevant, underlying mechanism of synergy for Cr or Mo with paromomycin.

The yeast membrane-transport proteins Sul1 and Sul2 are the sites at which chromate competes with sulphate for uptake^23^ and a *sul1Δ/sul2Δ* mutant is resistant to this competitive action^21^. Corroborating that the synergy with aminoglycosides involves inhibition of sulphate transport, synergy between chromate or molybdate and paromomycin was suppressed in the *sul1Δ/sul2Δ* mutant, even at elevated paromomycin concentrations (Fig. 3). Unlike results for the *cys3Δ/met15Δ* mutant, deletion of *SUL1* and *SUL2* was also effective in suppressing synergy between hygromycin B and DIDS.

### The translation error-rate is synergistically increased by combining aminoglycoside with sulphate transport inhibitor

**Figure 4:**
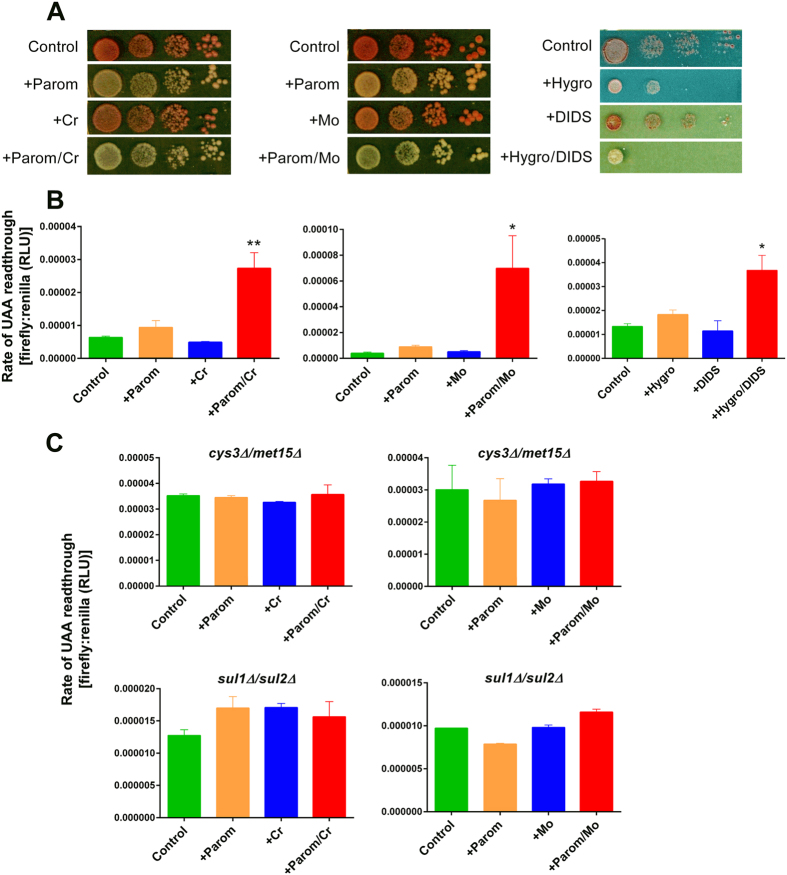
The translation error-rate is synergistically increased by combining paromomycin with sulphate mimetics. (**A**) *S. cerevisiae* L1494 (*ade1-14*) cells in 10-fold serial dilutions were spotted and incubated on YEPD agar supplemented with 25 μg ml^−1^ paromomycin, 50 μM chromate and/or 250 μM molybdate, or to YNB agar with 100 μg ml^−1^ hygromycin B and/or 1 mM DIDS. (**B**) Wild type yeast transformed with the dual-luciferase plasmid^36^ were exposed to 50 μg ml^−1^ aminoglycoside and/or 100 μM chromate, 500 μM molybdate, or 1 mM DIDS in YNB broth before determination of firefly and *Renilla* luciferase activities in protein extracts. The ratio of these activities indicates the level of translation read-through of the UAA stop codon separating the two ORFs. All values are means ±SEM from at least three independent determinations. RLU, relative light units. (**C**) The isogenic *cys3Δ/met15Δ* and *sul1Δ/sul2Δ* yeast double-deletants were treated as in (**B**). *p < 0.05 and **p < 0.01, according to one-way ANOVA.

Whereas chromate is thought to cause translation errors by limiting the availability of sulphur-containing amino acids for cognate tRNAs^16^,^21^,^24^, aminoglycosides like paromomycin are thought to induce a structural alteration in the small ribosomal subunit decoding centre^18^,^25^. Since parallel mechanisms like this which target a single phenotype are a common basis for drug-drug synergies, we tested whether the synergistic growth inhibition (Figs 1 and 2) was reflected by synergistic mistranslation. First we used a qualitative assay, based on translational read-through of a premature stop codon carried in the *ade1-14* allele of *S. cerevisiae* L1494^16^. Read-through suppresses the red pigmentation associated with this allele. Cr, Mo or DIDS alone had negligible effect on red-colony phenotype at the doses used, but in combination with aminoglycosides produced a slightly paler colour than aminoglycoside alone, suggesting read-through (Fig. 4A).

For a quantitative indication of mistranslation synergy, the rate of translational read-through of a UAA nonsense (stop) codon was monitored in a short-term dual-luciferase assay^16^. The plasmid borne construct used for this assay encodes a *Renilla* luciferase followed by a firefly luciferase with these ORFs separated by the UAA stop codon. The firefly luciferase should only be expressed following inappropriate read-through of the stop codon. Therefore, the measured ratio of the firefly to *Renilla* luciferase activities indicates the rate of mistranslation. The rate of read-through was increased ∼5-fold by a combination of Cr and paromomycin, in conditions where neither agent alone had a strong effect at the doses used (each agent alone causes read-through at higher doses) (Fig. 4B). A synergistic elevation of mistranslation rate was also obtained for the paromomycin-Mo and hygromycin-DIDS combinations. Consistent with the growth results (Fig. 3), synergistic mistranslation (by paromomycin and Cr or Mo) was abolished in a *cys3Δ/met15Δ* or *sul1Δ/sul2Δ* mutant (Fig. 4C). (We did not test mistranslation for DIDS with these mutants as the corresponding growth results were mixed; Fig. 3). The results are consistent with the hypothesis that combined targeting of translation fidelity causes synergistic growth inhibition by the sulphate mimetics combined with aminoglycoside.

### Sensitivity of pathogenic and food-spoilage fungi to combinatorial inhibition

**Figure 5:**
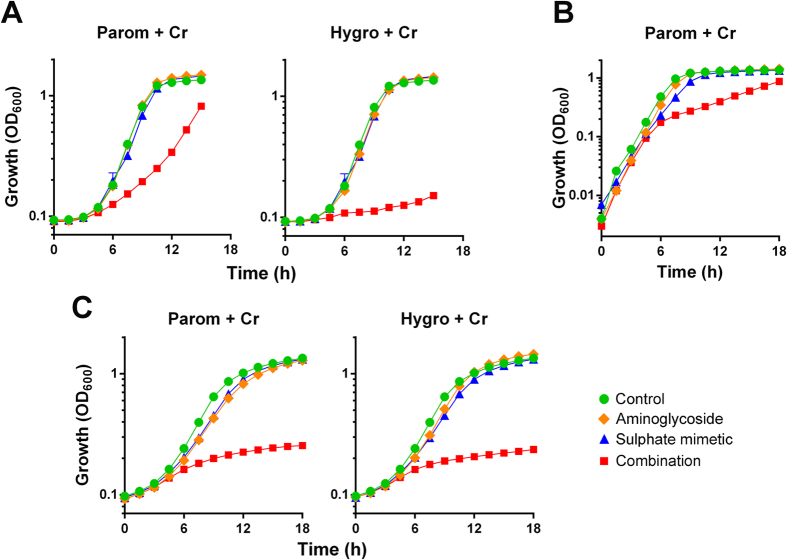
Synergistic growth inhibition of fungi pathogenic to humans. (**A**) Growth of *C. albicans* in YEPD broth supplemented with 200 μg ml^−1^ paromomycin, 10 μg ml^−1^ hygromycin B and/or 25 μM chromate. (**B**) Growth of *C. glabrata* in YEPD broth supplemented with 400 μg ml^−1^ paromomycin and/or 50 μM chromate. (**C**) Growth of *Cryptococcus neoformans* in YEPD broth supplemented with 12.5 μg ml^−1^ paromomycin, 0.625 μg ml^−1^ hygromycin B and/or 12.5 μM chromate. Data shown are replicates from two independent cultures ± SEM where these are larger than the symbol dimensions. The data for each condition are representative of at least two independent experiments performed on different days.

**Figure 6:**
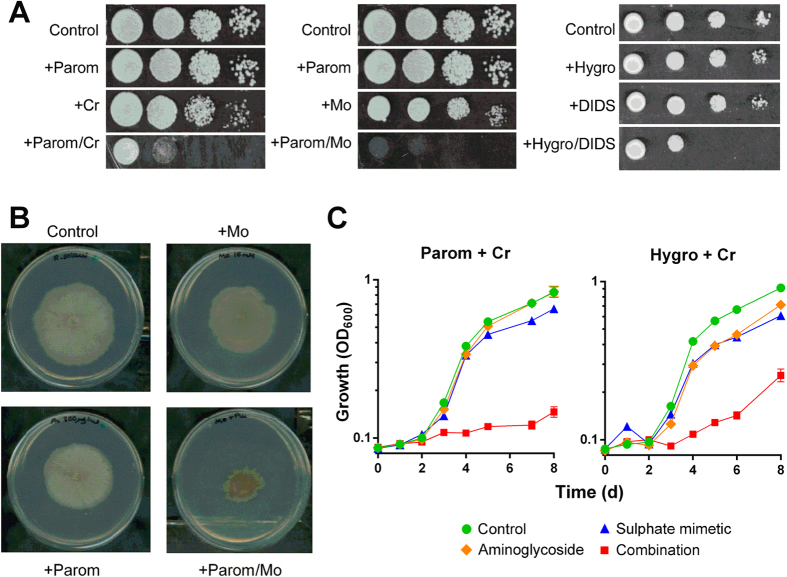
Synergistic growth inhibition of other problem fungi. (**A**) Growth of *Z. bailii* after spotting 10-fold serial dilutions of cell suspension on YEPD agar supplemented with 10 μg ml^−1^ paromomycin, 50 μM chromate and/or 1 mM molybdate, or to YNB agar with 10 μg ml^−1^ hygromycin B and/or 250 μM DIDS. (**B**) Growth of *R. solani* on PDA agar supplemented with 300 μg ml^−1^ paromomycin and 15 mM molybdate. (**C**) Growth of *Z. tritici* in PDB medium supplemented with 0.5 μg ml^−1^ paromomycin, 0.25 μg ml^−1^ hygromycin B and/or 10 μM chromate. The data for each condition are representative of at least two independent experiments performed on different days.

The effects of aminoglycoside in combinations with a sulphate mimetic were tested against key fungi of interest. Again, agents were supplied at doses which, individually, were near sub-inhibitory to each of the fungi. Accordingly, inclusion of either 25 μM chromate or 10 μg ml^−1^ hygromycin had no discernible inhibitory effect on growth of the human pathogen *Candida albicans* (Fig. 5A). However, growth yield was reduced ≥90% when the two agents were supplied in combination. Chromate also inhibited *C. albicans* in combination with paromomycin. Checkerboard analysis corroborated that these combinations decreased the MICs of the individual agents by ≥16-fold (Supplementary Fig. S2A). Molybdate was highly inhibitory to *C. albicans* in combination with paromomycin or hygromycin (Supplementary Fig. S2B). Sodium-bicarbonate and -orthovanadate were also tested, but did not produce synergy with aminoglycosides against *C. albicans* (data not shown). Some synergistic growth inhibition by paromomycin and Cr was also evident with another pathogenic *Candida* species, *C. glabrata*, although the effect on cell doubling time did not become apparent until mid-exponential growth (∼6 h) (Fig. 5B). Combinations of Cr with paromomycin or hygromycin (Fig. 5C) and to some extent streptomycin (Supplementary Fig. S2C) gave effective synergistic inhibition of the pathogenic yeast *Cryptococcus neoformans*. We did not detect obvious synergy between paromomycin and Cr against the filamentous fungi *Aspergillus fumigatus* (human pathogen) or *Botrytis cinerea* (plant pathogen), where the effect of the combination was not significantly different from that which could be accounted for by an additive effect of the individual treatments (Supplementary Fig. S3). Importantly, non-fungal test organisms were not sensitive to the synergy: neither the bacterium *E. coli* nor a human cell line exhibited synergistic inhibition by the combination (Supplementary Fig. S3; note the concentration of paromomycin alone that was just sub-inhibitory to *E. coli* was particularly low owing to this organism’s paromomycin hyper-sensitivity). There was synergistic growth inhibition of the food spoilage yeast *Zygosaccharomyces bailii* (Fig. 6A). Most dilutions of *Z. bailii* cell suspensions showed little growth on agar supplemented with paromomycin and chromate, at concentrations where neither agent alone had a marked growth effect. We additionally tested the molybdate-paromomycin and hygromycin-DIDS combinations against *Z. bailii,* with similar results. Synergistic growth inhibition of the fungal plant pathogen *Rhizoctonia solani* was also evident (Fig. 6B). Radial growth of *R. solani* from a central, point inoculum on agar was compromised by incorporation of paromomycin and molybdate into the medium, supplied at concentrations where neither individual agent had a marked growth-inhibitory effect. Similarly, growth yield of the plant pathogen *Zymoseptoria tritici* was synergistically inhibited by up to 90% using combinations of chromate (but not molybdate) with paromomycin or hygromycin B (but not streptomycin) (Fig. 6C). The results show that a number of important undesirable fungi are hyper-sensitive to targeting of translation fidelity using combinations of aminoglycoside with certain sulphate transport inhibitors.

## Discussion

Within a previous study, it was observed that paromomycin and chromate synergistically inhibit growth of the laboratory yeast *S. cerevisiae*^16^. The present results establish that other aminoglycosides besides paromomycin are effective in the combination and that impact on sulphate transport is the relevant property of chromate, as other sulphate-mimetics were also effective and appropriate mutants abolished the synergy. The evidence also indicates that amplification of translation error-rate is the mode of synergistic action. Moreover, certain major pathogenic and food spoilage fungi are susceptible to the combinations. We propose that combination targeting of translation fidelity offers a new potential strategy for managing such fungi.

Synergistic effects of more than one agent are commonly seen where they target a common process but by different mechanisms or pathways^26^. This appears to be the case here. Aminoglycosides are thought to promote translation errors by causing a structural alteration in the small ribosomal subunit decoding centre, allowing entry of near-cognate tRNAs^18^,^25^. On the other hand, chromate (and presumably related sulphate mimetics) compete with sulphate for uptake, leading to starvation for sulphur-containing amino acid acids and mistranslation^21^,^23^. This dual targeting of translation fidelity was effective in synergistically increasing translation error-rate and decreasing growth rate. Such types of combination are particularly useful for antimicrobial application as they decrease not only the risk of resistance evolution^8^,^27^ but the synergy also allows much lower amounts of active agents to be used. Translation fidelity is a particularly attractive target as its effects are not simply due to loss of essential (protein) functions, but also gain of toxic function through formation of inhibitory protein aggregates^16^.

The actual concentrations that were effective here varied depending on the aminoglycoside used, the sulphate-mimetic, the fungus and the growth medium. Clearly, the lower the effective concentrations, the more desirable in any application as this lessens potential concerns over non-specific toxicity or cost; even when the agents are relatively cheap, as is largely the case here. As certain of the agent types used here already have had some application singly, decreasing their effective concentrations in a combined approach can only alleviate any concerns over dosage levels. In several cases, concentrations that gave little or no inhibition individually were sufficient to give close to 100% growth inhibition when used in combination; with MICs decreased ≥8-fold in the combinations, some synergistic growth inhibition was still achievable at considerably lower doses in many of these examples. It is also important to note that the screen of aminoglycosides and sulphate mimetics was not exhaustive and other analogues effective in the combination with different MICs are likely to be found. This is particularly true for any non-competitive inhibitors of the relevant sulphate transporters which, being non-competitive, may require lower doses to be effective than the competitive inhibitors used here. Finally, it is important to note that the *in vitro* conditions used for the present tests were valuable for demonstrating the synergy against different fungi and for elucidating mode-of-action, but effective concentrations are likely to vary considerably in different plant or animal tissues and other environments requiring fungal control^28^,^29^.

Specificity for target organisms is a key consideration for any antimicrobial treatment. Like other agents, aminoglycosides and sulphate-mimetics may of course ultimately inhibit any organism given sufficiently high doses. Relatively low concentrations of aminoglycosides are commonly needed to inhibit Gram negative bacteria, the usual target organisms for these antibiotics. However, neither *E. coli* nor a human cell line exhibited sensitivity to the synergistic action of aminoglycoside in combination with sulphate-mimetic. The potential impact of sulphate-mimetics (or sulphate starvation) on translation error in these organisms has not been investigated to our knowledge. Given that sulphate uptake is not necessary for supply of methionine (essential) or cysteine (conditionally essential) in humans, there is no rationale for expecting sulphate-mimetics to provoke mistranslation in this case. In other words, a mechanistic requirement for the synergy to work in fungi is absent in humans, consistent with the synergistic activity being target specific. One apparent inconsistency among the mechanism-of-action data was the observation that deletion of *SUL1*+*SUL2* but not of *CYS3*+*MET15* suppressed synergy between hygromycin and DIDS in yeast. The most straightforward explanation is that DIDS also interferes with Cys or Met transport, which would preclude these supplements from rescuing a blockage by DIDS of Sul1/Sul2-dependent sulphate availability. Given that DIDS is a general anion exchange inhibitor, in contrast to chromate and molybdate tested here specifically as sulphate mimetics, it is reasonable to expect that DIDS would be the most likely to exert non-specific effects, e.g. on processes impacting Cys or Met transport^30^.

Among the fungi tested, there was variation in the combinations that were most effective and the relative degree of sensitivity attained. All five of the yeasts showed sensitivity, in contrast to only *R. solani* and *Z. tritici* of the four filamentous fungi tested with the paromomycin and chromate combination. As the only basidiomycetes tested, *R. solani* and *C. neoformans* are less closely related to the other yeasts phylogenetically than the combination-resistant filamentous fungi *B. cinerea* or *A. fumigatus* (ascomycetes)^31^. All of the fungi tested express orthologues of the yeast Sul1 and/or Sul2 proteins, the predicted site of action of the sulphate mimetics^21^. Orthologue identity with the yeast protein sequences is slightly greater for *R. solani* (43 and 44% identity) than *A. fumigatus* or *B. cinerea* (38–41% identity). On this basis, of the four filamentous fungi it is *R. solani* and *Z. tritici* that cluster most closely with the combination-sensitive yeasts (Supplementary Fig. S4), suggesting that sequence similarity to the yeast Sul1/Sul2 proteins could provide a predictor of sensitivity.

The potential of translation fidelity as a target for combinatorial inhibition of fungal growth is exemplified by the present results for *Z. bailii.* This organism is notoriously resistant to agents like weak acids, commonly used for control of food spoilage by yeasts^32^. *Z. bailii* was hyper-sensitive to inhibition in this study, requiring comparatively low concentrations of aminoglycoside in the combinations to achieve synergy. Whereas regulatory constraints would preclude the use of many of the agents described here to control *Z. bailii* in foods intended for human consumption, such constraints are less of an issue for other applications in fungal control, such as building-mould or fungal plant disease; the wheat pathogen *Z. tritici* was also hyper-sensitive to synergy at low doses. Aminoglycosides are already approved and widely used as anti-infectives in humans. Moreover, as this work establishes translation fidelity as a potential combinatorial target, it opens the door to discovery of alternative agents that can be substituted to achieve similar (or better) synergy and more-tailored activity spectra and safety profiles.

## Methods

### Fungal strains and maintenance

Mode of action studies were performed primarily with *Saccharomyces cerevisiae* BY4743 or, for experiments involving the *cys3Δ*/*met15Δ* and *sul1Δ*/*sul2Δ* mutants^21^, the isogenic haploid BY4741. *S. cerevisiae* L1494 (*ade1-14*) was used for red/white mistranslation assays (below). Other fungi were the yeasts *Candida albicans* SC5314*, Candida glabrata* BG2, *Cryptococcus neoformans* 1841 and *Zygosaccharomyces bailii* NCYC 1766, and the filamentous fungi *Aspergillus fumigatus* Af293, *Botrytis cinerea* SAR109940, *Rhizoctonia solani* AG2-1 1939 and *Zymoseptoria tritici*. The yeasts were routinely grown and maintained on YEPD medium: 1% (w/v) yeast extract (Oxoid), 2% (w/v) peptone (Oxoid), 2% (w/v) glucose. Where specified, yeasts were cultured in YNB medium [0.69% yeast-nitrogen base without amino acids (Formedium), 2% (w/v) D-glucose], supplemented as required with amino acids or nucleobases to complement auxotrophies or for plasmid selection. Where necessary, media were solidified with 2% (w/v) agar (Sigma-Aldrich, St. Louis, MO). The filamentous fungi were routinely maintained and grown on potato dextrose agar (PDA; Oxoid) except *A. fumigatus* which was on Aspergillus complete medium (ACM)^33^.

### Chemicals

With the exception of hygromycin B (Panreac Applichem), all chemicals were from Sigma-Aldrich: paromomycin, streptomycin, Na_2_CrO_4_, Na_2_MoO_4_, Na_3_VO_4_, 4,4′-diisothio-cyanatostilbene-2,2′-disulphonate (DIDS), sodium malonate, sodium oxalate, NaHCO_3_, Na_2_SeO_4_, Na_2_S_2_O_3_, Na_2_WO_4_, Na_2_S_4_O_6_. With the exception of DIDS (in 0.1 M potassium bicarbonate), stock solutions of all chemicals used to inhibit growth were prepared in distilled water and added to growth media at the specified final concentrations. All stock solutions were filter-sterilized before additions to media.

### Inhibition assays in broth

Yeasts were inoculated from YEPD plates to YEPD broth and cultured overnight at 30 °C, 120 rev min^−1^. Overnight cultures were diluted to OD_600_~0.5 and cultured for a further 4 h in fresh YEPD before dilution of these experimental cultures to OD_600_~0.01 or ~0.1 in the same medium. Aliquots (300 μl) of the diluted culture plus any chemical supplements (see above), as specified, were transferred to 48-well plates (Greiner Bio-One). OD_600_ was monitored continuously in a BioTek Powerwave-XS microplate spectrophotometer, with shaking at 30 C. *Escherichia coli* was tested in a similar way but with growth in LB broth and at 37 C. Spores of *Z. tritici* were inoculated from PDA plates to potato dextrose broth (PDB; Fluka) (20,000 spores ml^−1^). Aliquots (150 μl) of the diluted culture plus any chemical supplements, as specified, were transferred to 96-well plates (Greiner Bio-one) and cultured over 8 d at 24 °C, 120 rev min^−1^. OD_600_ was monitored every day in a BioTek EL800 microplate spectrophotometer. Human TE671 cells were cultured in DMEM supplemented with 10% foetal bovine serum, L-glutamine (2 mM), penicillin (100 U ml^−1^), streptomycin (100 μg ml^−1^) in 25 cm^2^ cell culture flasks, 36.5 °C, 5% oxygen. Cells were detached with trypsin/EDTA and washed in 10 ml DMEM. Then 100 ul of cell suspension (in DMEM without antibiotics) were dispensed to 5000 cells/well in a 96-well plate. After 24 h, paromomycin or chromate were added as specified. After a further 24 h, 10 μl of CCK-8 reagent (Sigma) were added to each well. After 4 h incubation, formazan production was determined at 450 nm using a BioTek EL800 microplate spectrophotometer.

### Checkerboard assays

All culturing and preparation for checkerboard assays adhered to EUCAST guidelines^34^,^35^ with the exception that YEPD broth at 30 C was necessary to support adequate growth of both *S. cerevisiae* and *C. albicans*. Five colonies from PDA plates grown at 30 C for ∼20 h were each suspended in YEPD and added with solutions of inhibitors as specified in 96-well microdilution plates to give final suspensions of 200 μl per well at cell densities of ∼1.5 × 10^5^ ml^−1^. The inoculated plates were incubated statically for 24 h at 30 C before measurement of OD_600_ with a BioTek EL800 microplate spectrophotometer. After subtraction of the background reading from readings for the rest of the wells, percentage growth for each condition was calculated relative to control growth in the absence of added inhibitors. Fractional inhibitory concentrations (FIC) were calculated as described^20^.

### Growth inhibition assays on solid medium

For qualitative growth-inhibition assays with *Z. bailii* on solid medium, experimental cultures prepared as described above were adjusted to OD_600_∼2.0, 0.2, 0.02, 0.002, 0.0002 and the dilution series spotted (4 μl) on to YEPD agar. Images were captured after 2 d growth at 30 C. For growth-inhibition assays on solid medium with filamentous fungi, circular sections of ~0.5 cm diameter were excised from cultures on PDA or ACM agar, and transferred to the centre of fresh plates. Images were captured after 3 d growth at 28 C and the total mycelial area determined using ImageJ and GIMP2 software. Where specified, chemical supplements were included in the solid media.

### Mistranslation assays

For qualitative determination of mistranslation, *S. cerevisiae* L1494 (*ade1-14*) was spotted as described above to either YEPD agar, or YNB agar in the case of DIDS/hygromycin (using YNB supplemented with 5 μg ml^−1^ adenine rather than the standard 20 μg ml^−1^). Images were captured for red versus white colony-colour comparisons after 2 d (YEPD) or 3 d (YNB) growth at 30 C. For quantitative determination of mistranslation, *S. cerevisiae* was transformed with a dual luciferase reporter plasmid encoding firefly and *Renilla* luciferases, the relative protein activities of which indicate the level of translational read-through of the UAA stop codon separating the two ORFs^16^,^36^. The organism was cultured as above in YNB broth appropriate for plasmid selection. Cells were exposed to specified agents for 3 h, before preparation of protein extracts and determination of the two luciferase activities as described previously^16^.

## Additional Information

**How to cite this article**: Moreno-Martinez, E. *et al.* Novel, Synergistic Antifungal Combinations that Target Translation Fidelity. *Sci. Rep.* **5**, 16700; doi: 10.1038/srep16700 (2015).

## Supplementary Material

Supplementary Information
