# Supplementary material for: Novel, Synergistic Antifungal Combinations that Target Translation Fidelity
Source: Sci Rep. 2015 Nov 17;5:16700. doi: 10.1038/srep16700 (PMC4648087; doi:10.1038/srep16700)
Supplement: Supplementary Information [file srep16700-s1.pdf]

SUPPLEMENTARY INFORMATION

Paper title: **Novel, Synergistic Antifungal Combinations that Target Translation Fidelity**

Elena Moreno-Martinez, Cindy Vallieres, Sara L. Holland, Simon V. Avery

School of Life Sciences

University of Nottingham

University Park

Nottingham NG7 2RD, UK

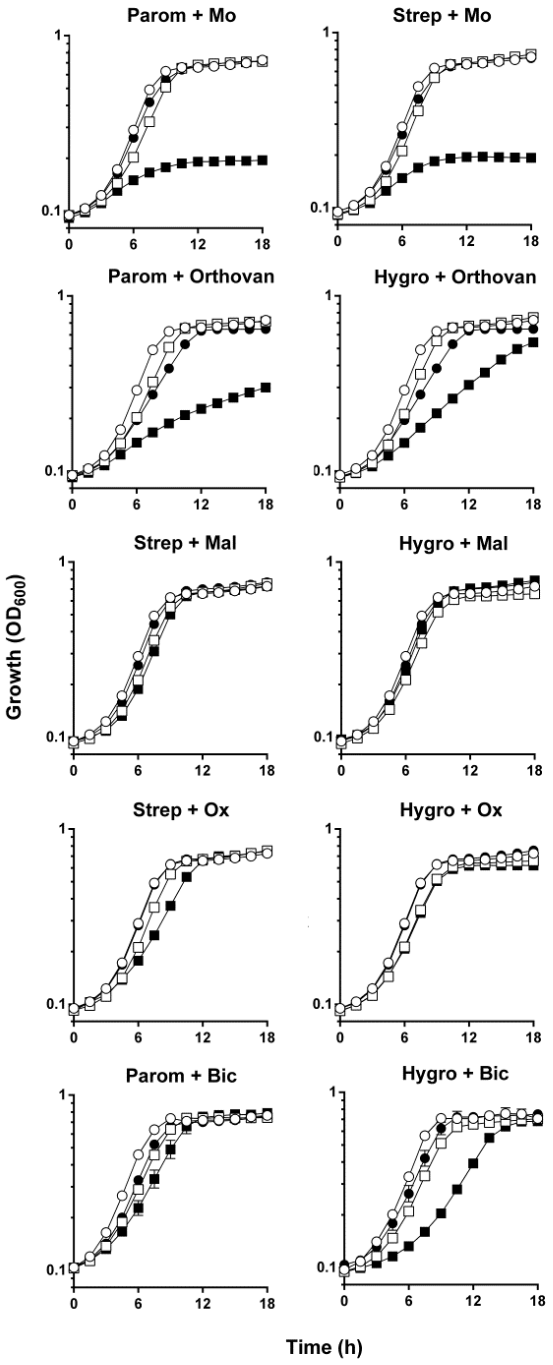

Figure S1. Effects of additional combinations of aminoglycosides and sulphate transport inhibitors on yeast growth. Growth of *S. cerevisiae* was monitored in YEPD supplemented with different aminoglycosides in combination with different sulphate transport inhibitors (the most effective synergistic combinations are shown in Figures 1 and 2, main paper). ○, control; □, +aminoglycoside; ●, +sulphate transport inhibitor; ■, +combination. The agents and doses were: paromomycin (Parom), 200-250 μg ml^{-1}; streptomycin (Strep), 30 mg ml^{-1} (the highest soluble dose attainable); hygromycin B (Hygro), 10 μg ml^{-1}; molybdate (Mo), 1 mM, orthovanadate (Orthovan), 1 mM; malonate (Mal), 50 mM (the highest sub-inhibitory dose tested); oxalate (Ox), 5 mM (the highest soluble dose attainable); bicarbonate (Bic), 7.5 mM.

A

Parom (mg/ml)								
51.2	79	70	-1	-5	-3	-4	-2	-2
25.6	105	29	0	-5	-5	-1	-4	-3
12.8	90	2	1	-4	1	3	2	-3
6.4	96	-7	-7	-6	-7	-5	-4	-5
3.2	90	-6	-7	-5	-6	-4	-4	-2
1.6	94	-4	-4	-2	-2	-2	-1	0
0.8	102	-1	-2	-1	-1	0	0	0
0	100	105	108	95	85	45	18	5
Cr (μM)	0	25	50	100	200	400	800	1600

Hygro (μg/ml)								
51.2	0	0	0	0	0	0	0	1
25.6	1	1	0	1	1	1	1	1
12.8	8	0	0	0	0	0	0	0
6.4	63	0	0	0	0	0	0	0
3.2	85	0	0	0	0	0	0	0
1.6	95	1	0	1	0	0	0	0
0.8	109	1	0	0	0	0	0	0
0	100	108	107	87	82	45	18	4
Cr (μM)	0	25	50	100	200	400	800	1600

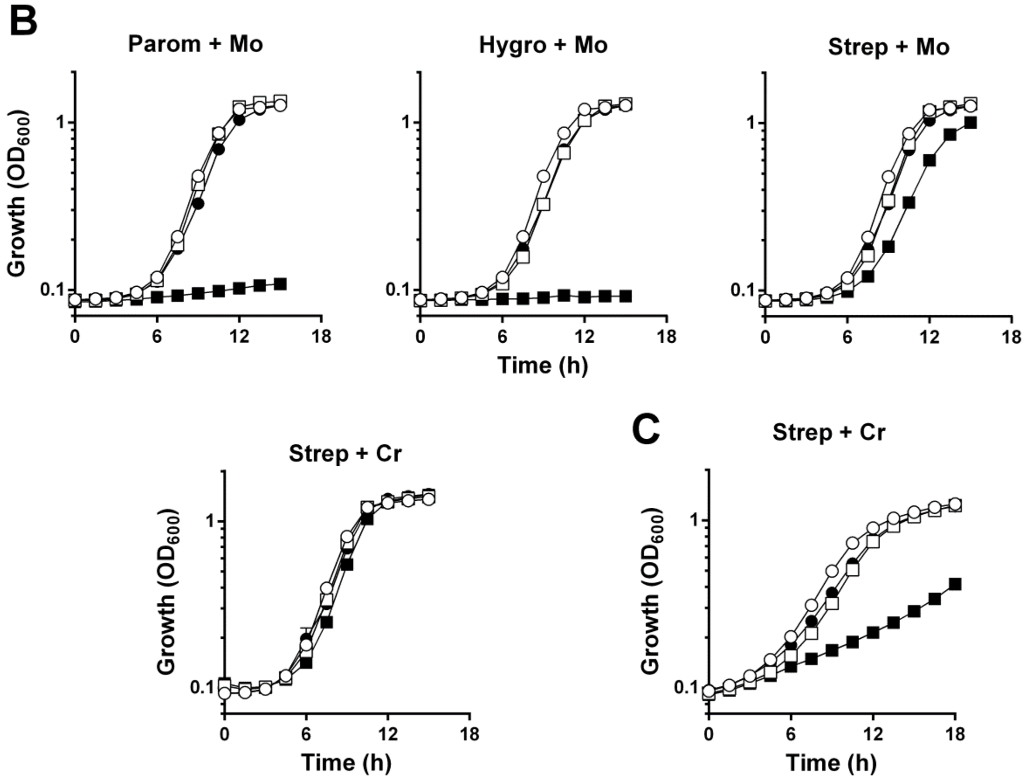

Figure S2. Effects of additional combinations of aminoglycosides and sulphate-mimetics on growth of human pathogens. A. Checkerboard assays with *C. albicans* at the indicated concentrations, according to EUCAST procedure. The growth values within the boxes are percentages of control growth (OD) determined in the absence of aminoglycoside or Cr. Shaded boxes indicate ≥50% of control growth. B. Growth of *C. albicans* in YEPD broth supplemented with 200 μg ml^{-1} paromomycin, 30 mg ml^{-1} streptomycin, 10 μg ml^{-1} hygromycin B, 25 μM chromate and/or 10 mM molybdate. ○, control; □, +aminoglycoside; ●, +sulphate transport inhibitor; ■, +combination. C. Growth of *C. neoformans* with 500 μg ml^{-1} streptomycin and 12.5 μM chromate. Other details are as for panel B. Other effective synergistic combinations for *C. albicans* and *C. neoformans* are shown in Figure 5, main paper.

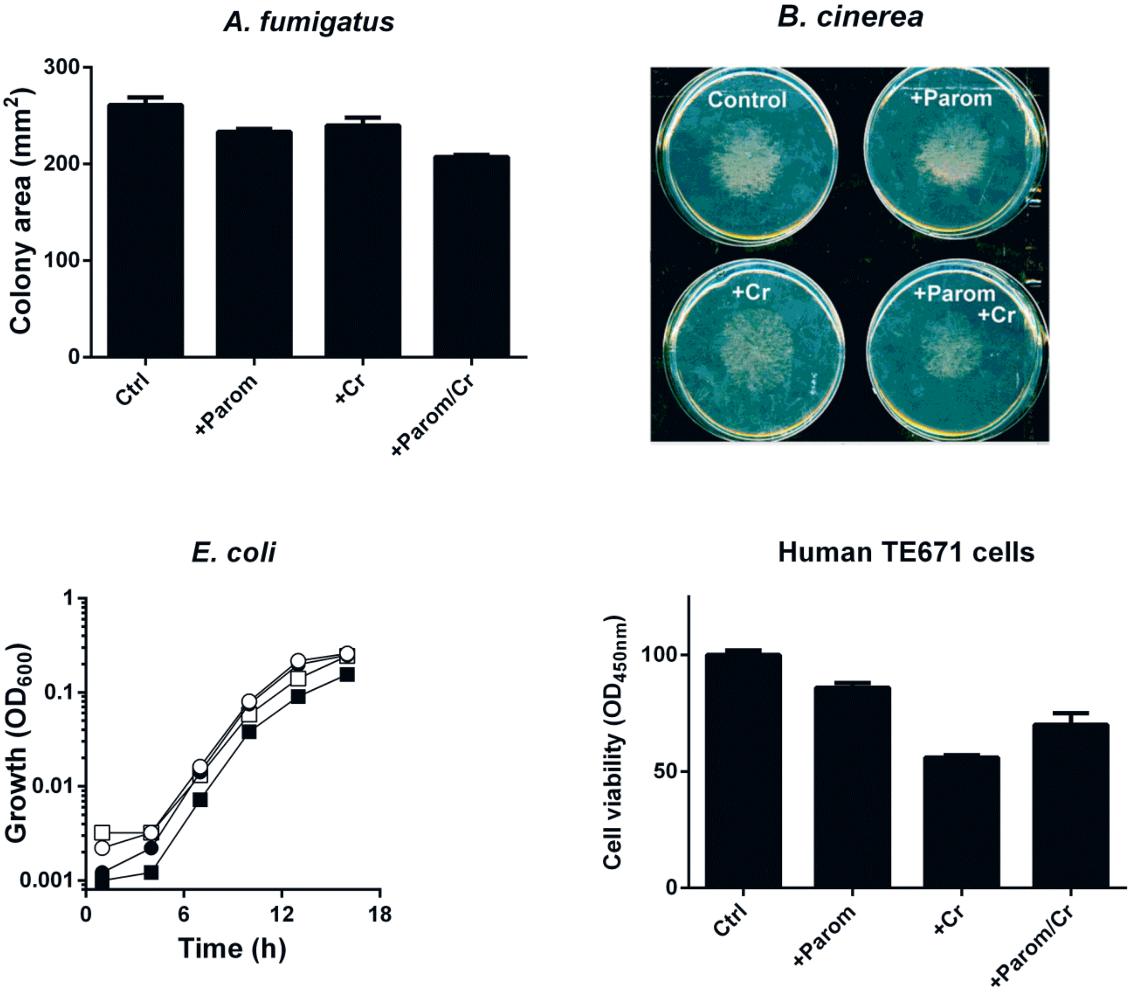

Figure S3. Organisms not susceptible to synergistic growth inhibition by paromomycin and chromate. Growth according to colony area of *A. fumigatus* on ACM agar supplemented as indicated with 200 μg ml^{-1} paromomycin and/or 50 μM chromate; *B. cinerea* on PDA agar supplemented with 50 μg ml^{-1} paromomycin and/or 50 μM chromate (absence of synergy was confirmed in quantitative broth assays, not shown); *E. coli* in LB broth supplemented without (○) or with 1 μg ml^{-1} paromomycin (□), 15 μM chromate (●), or both agents (■); Human cells were incubated in DMEM broth supplemented with 1 mg ml^{-1} paromomycin and/or 10 μM chromate and relative viability was estimated from tetrazolium reduction activity. Data are representative of more than one independent experiment performed on different days.

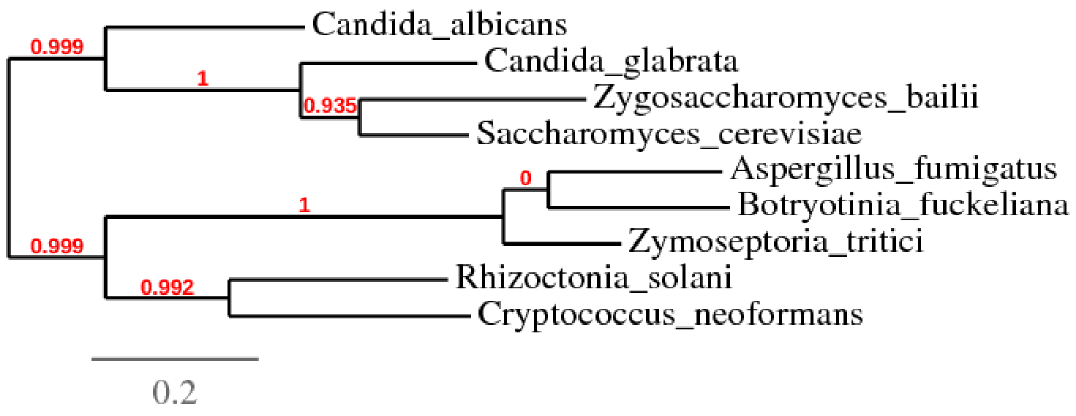

Figure S4. Phylogenetic tree according to relatedness of orthologues from different fungi to Sul1p from *S. cerevisiae.* The relevant NCBI accession numbers are KGU30045.1 (*C. albicans*), XP_449305.1 (*C. glabrata*), XP_566490.1 (*C. neoformans*), KEP54943.1 (*R. solani*), XP_001559023.1 (*Botryotinia fuckeliana (B. cinerea),* XP_750251.1 (*A. fumigatus*), CDH12594.1 (*Z. bailii*), XP_001584768.1 (*Z. tritici*). The tree was constructed using the programme at http://phylogeny.lirmm.fr/phylo cgi/index.cgi . The Gblocks programme was used to eliminate poorly aligned positions and divergent regions. Branch lengths are proportional to the number of substitutions per site.
